# Non contiguous-finished genome sequence and description of *Senegalemassilia anaerobia* gen. nov., sp. nov.

**DOI:** 10.4056/sigs.3246665

**Published:** 2013-02-25

**Authors:** Jean-Christophe Lagier, Khalid Elkarkouri, Romain Rivet, Carine Couderc, Didier Raoult, Pierre-Edouard Fournier

**Affiliations:** 1 Aix-Marseille Université, URMITE, Faculté de médecine, Marseille, France

**Keywords:** *Senegalemassilia anaerobia*, genome

## Abstract

*Senegalemassilia anaerobia* strain JC110^T^ sp.nov. is the type strain of *Senegalemassilia anaerobia* gen. nov., sp. nov., the type species of a new genus within the *Coriobacteriaceae* family, *Senegalemassilia* gen. nov. This strain, whose genome is described here, was isolated from the fecal flora of a healthy Senegalese patient. *S. anaerobia* is a Gram-positive anaerobic coccobacillus. Here we describe the features of this organism, together with the complete genome sequence and annotation. The 2,383,131 bp long genome contains 1,932 protein-coding and 58 RNA genes.

## Introduction

*Senegalemassilia anaerobia* strain JC110^T^ (= CSUR P147 = DSMZ 25959) is the type strain of *S. anaerobia* gen. nov., sp. nov. This bacterium was isolated from the feces of a healthy Senegalese patient. It is a Gram-positive, anaerobic, indole-negative coccobacillus. Classically, the polyphasic taxonomy is used to classify the prokaryotes by associating phenotypic and genotypic characteristics [1]. Culturomics is a new subfield of genomics aimed at studying the microbial repertoire of the gut, and has already lead to the isolation of many new bacterial species [2]. In parallel, as more than 3,000 bacterial genomes have been sequenced so far, we proposed to integrate genomic data in descriptions of new bacterial species [3-15].

The family *Coriobacteriaceae* was created in 1997, in the class *Actinobacteria*, and currently contains 13 genera of anaerobic Gram-positive members of the normal intestinal microbiota from humans and animals [16-28]. Among them, *Gordonibacter* and *Paraeggherthella* have occasionally been isolated from Crohn’s disease specimens [26].

Here we present a summary classification and a set of features for *S. anaerobia* gen. nov., sp. nov. strain JC110^T^ together with the description of the complete genomic sequencing and annotation. These characteristics support the circumscription of the genus *Senegalemassilia* and the species *S. anaerobia*.

## Classification and features

A stool sample was collected from a healthy 16-year-old male Senegalese volunteer patient living in Dielmo (rural village in the Guinean-Sudanian zone in Senegal), who was included in a research protocol. Written assent was obtained from this individual. No written consent was needed from his guardians for this study because he was older than 15 years old (in accordance with the previous project approved by the Ministry of Health of Senegal, the assembled village population, and as published elsewhere [28]. Both this study and the assent procedure were approved by the National Ethics Committee of Senegal (CNERS) and the Ethics Committee of the Institut Fédératif de Recherche IFR48, Faculty of Medicine, Marseille, France (agreement numbers 09-022 and 11-017). Several other new bacterial species were isolated from this specimen using various culture conditions, including the recently described *Anaerococcus senegalensis, Alistipes senegalensis, Alistipes timonensis, Peptoniphilus timonensis, Clostridium senegalense, Paenibacillus senegalensis* and *Bacillus timonensis*, *Herbaspirillum massiliense, Kurthia massiliensis, Brevibacterium senegalense, Aeromicrobium massiliense and Cellulomonas massiliensis*[3-15].

**Figure 1:**
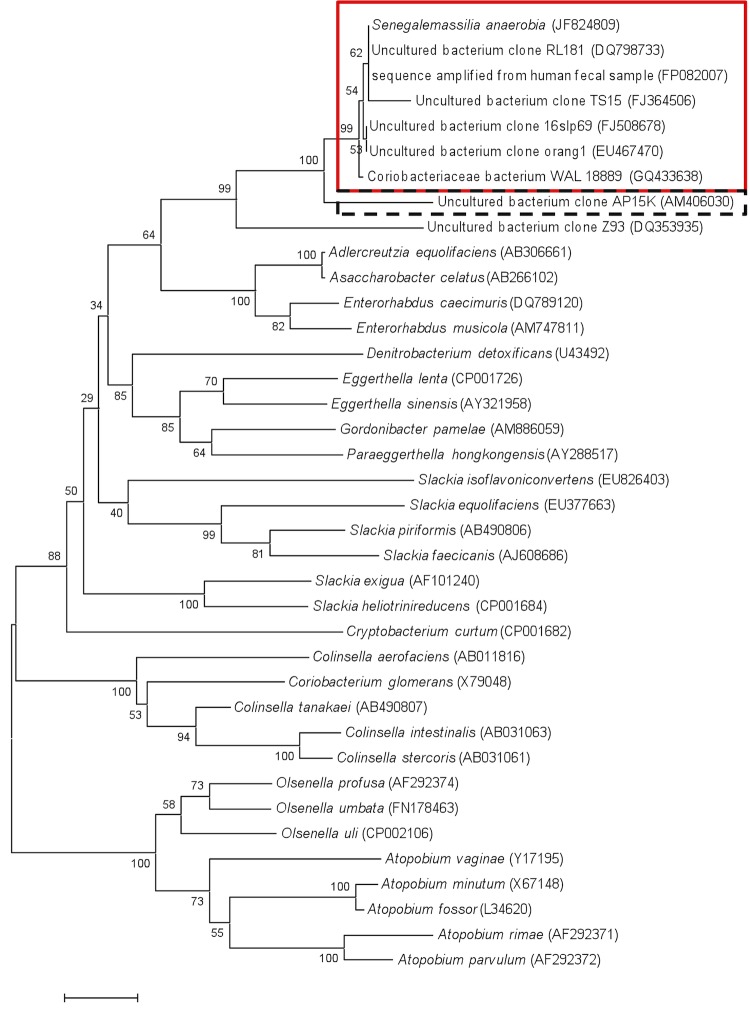
Phylogenetic tree highlighting the phylogenetic position of *Senegalemassilia anaerobia* strain JC110^T^ relative to other type strains within the *Coriobacteriaceae* family. GenBank accession numbers are indicated in parentheses. Sequences were aligned using CLUSTALW, and phylogenetic inferences were made using the maximum-likelihood method within the MEGA software. Numbers at the nodes are percentages of bootstrap values (500 repetitions) to generate a majority consensus tree. The scale bar indicates a 1% nucleotide sequence divergence. The red square groups sequences that exhibit degrees of similarity > 99% with *S. anaerobia* (same species), whereas that in the dashed-line square is 97.2% similar (same genus).

The fecal specimen was preserved at -80°C after collection and sent to Marseille. Strain JC110^T^ (Table 1) was isolated in February 2011. The stool was preincubated for 5 days in a blood culture bottle, and then inoculated onto 5% sheep blood agar and incubated in anaerobic atmosphere at 37°C. The strain exhibited a nucleotide sequence similarity with members of the *Coriobacteriaceae* ranging from 85.3% with *Atopobium parvulum* to 92.4% with *Enterorhabdus mucosicola* (Figure 1). This value was lower than the 95% 16S rRNA gene sequence threshold recommended by Stackebrandt and Ebers to delineate a new genus [33]. By comparison to the NR database, strain JC110 ^T^ also exhibited nucleotide sequence similarities greater than 99% with uncultured bacterial clones detected in metagenomic studies of the human gut flora. These bacteria are most likely classified within the same species as strain JC110 (Figure 1).

**Table 1:** Classification and general features of *Senegalemassilia anaerobia* strain JC110^T^ according to the MIGS recommendations [29]

**MIGS ID**	**Property**	**Term**	**Evidence code^a^**
	Current classification	Domain *Bacteria*	TAS [30]
		Phylum *Actinobacteria*	TAS [31]
		Class *Actinobacteria*	TAS [16]
		Order *Coriobacteriales*	TAS [16,32]
		Family *Coriobacteriaceae*	TAS [16,32]
		Genus *Senegalemassilia*	TAS
		Species *Senegalemassilia anaerobia*	IDA
		Type strain JC110^T^	
	Gram stain	positive	IDA
	Cell shape	coccobacillus	IDA
	Motility	motile	IDA
	Sporulation	nonsporulating	IDA
	Temperature range	mesophile	IDA
	Optimum temperature	37°C	IDA
MIGS-6.3	Salinity	unknown	IDA
MIGS-22	Oxygen requirement	anaerobic	IDA
	Carbon source	unknown	
	Energy source	unknown	
MIGS-6	Habitat	human gut	IDA
MIGS-15	Biotic relationship	free living	IDA
MIGS-14	Pathogenicity Biosafety level Isolation	unknown 2 human feces	
MIGS-4	Geographic location	Senegal	IDA
MIGS-5	Sample collection time	September 2010	IDA
MIGS-4.1	Latitude	13.7167	IDA
MIGS-4.1	Longitude	– 16.4167	IDA
MIGS-4.3	Depth	Surface	IDA
MIGS-4.4	Altitude	51 m above sea level	IDA

Evidence codes - IDA: Inferred from Direct Assay; TAS: Traceable Author Statement (i.e., a direct report exists in the literature); NAS: Non-traceable Author Statement (i.e., not directly observed for the living, isolated sample, but based on a generally accepted property for the species, or anecdotal evidence). These evidence codes are from the Gene Ontology project [33]. If the evidence is IDA, then the property was directly observed for a live isolate by one of the authors or an expert mentioned in the acknowledgements.

**Figure 2:**
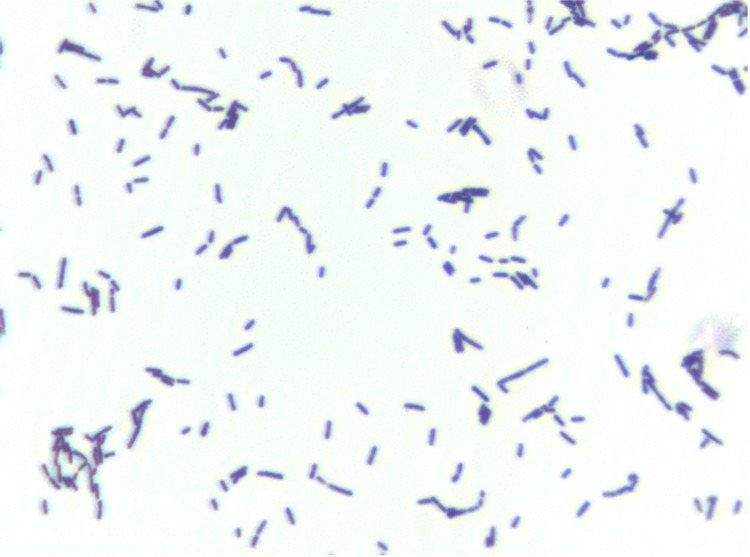
Gram staining of *S. anaerobia* strain JC110^T^

**Figure 3:**
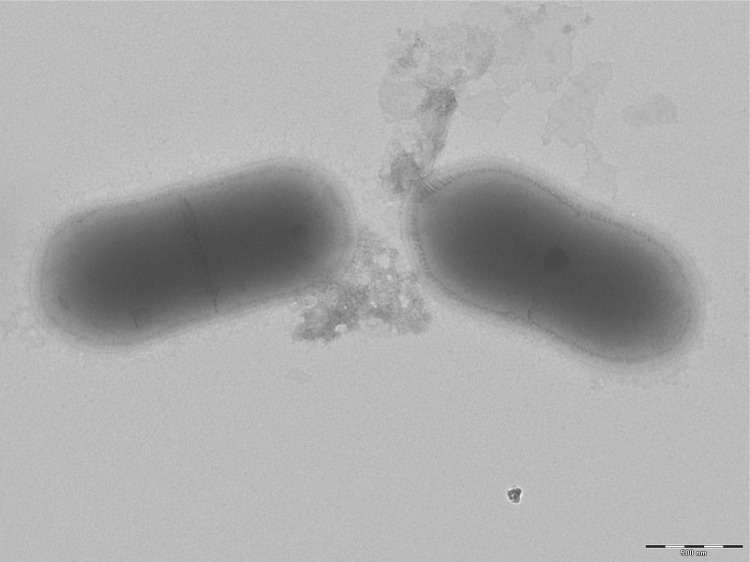
Transmission electron microscopy of *S. anaerobia* strain JC110^T^, using a Morgani 268D (Philips) at an operating voltage of 60kV.The scale bar represents 900 nm. Length and diameter were measured about 10 different bacteria.

Different growth temperatures (25, 30, 37, 45°C) were tested; no growth occurred at 25°C or 45°C, weak growth occurred at 30°C, optimal growth was observed at 37°C. Colonies were transparent and smooth with 0.5 mm in diameter on blood-enriched Columbia agar and Brain Heart Infusion (BHI) agar. Growth of the strain was tested under anaerobic and microaerophilic conditions using GENbag anaer and GENbag microaer systems, respectively (BioMérieux), and in the presence of air, of 5% CO2 and in aerobic conditions. Growth only occurred under anaerobic conditions. A motility test was positive. Cells grown on agar appear as Gram-positive coccobacilli (Figure 2) and have a diameter ranging from 0.62 to 0.76 µm (mean of 0.70 µm) and a length ranging from 1.36 to 1.73 µm (mean of 1.56 µm)(Figure 3).

Strain 110^T^ exhibited neither catalase nor oxidase activities. In the API Rapid ID 32A system, positive reactions were obtained for arginine dihydrolase, and nitrate reduction. A weak reaction was obtained for alkanine phosphatase. In the API ZYM system, positive reaction was observed for Naphthlol-AS-BI-phosphohydrolase and a weak reaction was observed for alkaline phosphatase and acid phosphatase. Negative reactions were observed for alkaline phosphatase, esterase, esterase lipase, lipase, leucine arylamidase, valine arylamidase, cystine arylamidase, trypsin, α-chymotrypsin, α-galactosidase, β-galactosidase, β-glucuronidase, α-glucosidase, β-glucosidase, N-acetyl- β-glucosaminidase, α-mannosidase and α-fucosidase. In the API 50CH system, all reactions were negative. *S. anaerobia* is susceptible to amoxicillin, imipenem, metronidazole and gentamicin but resistant to trimethoprim/sulfamethoxazole.

The comparisons with genera of the *Coriobacteriaceae* family are summarized in Table 2. *Senegalemassilia anaerobia* JC110^T^ shares motility with *Gordonibacter pamelae*,in contrast with *Adlercreutzia equolifaciens, Enterorhabdus mucosicola, Eggerthela sinensis* and *Collinsella aerofaciens*. In contrast with *Collinsella aerofaciens*, *Senegalemassilia anaerobia* was asaccharolytic. Among these species, JC110^T^ revealed a positive reaction for nitrate reductase. Lastly, we observed within the members of *Coriobacteriaceae* family a large heterogeneity of DNA G+C content ranging from 60% to 66.5% [Table 2].

**Table 2:** Differential characteristics of six members of the *Coriobacteriaceae* family^†^

Properties	*Senegalemassilia* *anaerobia*	*Adlercreutzia equolifaciens*	*Enterorhabdus mucosicola*	*Eggerthella sinensis*	*Gordonibacter pamelae*	*Collinsella aerofaciens*
Cell morphology	Coccobacilli	Coccobacilli	Rod	Rod	Coccobacilli	Chains of coccid cells
Oxygen requirement	Obligately anaerobic	Obligately anaerobic	Obligately anaerobic	Obligately anaerobic	Obligately anaerobic	Obligately anaerobic
Motility	+	–	–	–	+	–
Spore- formation	–	–	–	–	–	+
Production of						
Alkaline phosphatase	W	Na	Na	–	–	Na
Catalase	–	Na	–	+	+	Na
Oxidase	–	Na	–	Na	Na	Na
Nitrate reductase	+	–	Na	–	–	Na
Urease	–	–	Na	–	–	Na
Indole production	–	Na	–	–	–	Na
β-galactosidase	–	Na	Na	–	–	Na
β-glucosidase	–	–	Na	–	–	Na
Arginine arylamidase	–	+	Na	+	–	Na
Arginine dihydrolase	+	+	Na	+	+	Na
Leucine arylamidase	–	+	Na	–	–	Na
Acid from						
Glucose	–	–	Na	–	–	+
Arabinose	–	–	Na	–	–	–
Ribose	–	–	Na	Na	–	–
Mannose	–	–	Na	–	Na	+
Maltose	–	–	Na	Na	Na	+
Mannitol	–	–	Na	Na	Na	–
Trehalose	–	–	Na	–	–	–
Cellobiose	–	–	Na	Na	Na	–
Galactose	–	–	Na	Na	Na	+
Fructose	–	–	Na	Na	Na	+
G+C content (mol%)	60.9	64.1 to 66.5	64.2	64.9-65.6	66.4	60-61
Habitat	human gut	human and rat intestine	mouse intestine	human bacteremia	human colon Crohn’s disease	human samples

^†^*Senegalemassilia anaerobia strain* JC110^T^, *Adlercreutzia equolifaciens* strain FCJ-B9^T^, *Enterorhabdus mucosicola* strain Mt1B8^T^, *Eggerthella sinensis* HKU14, *Gordonibacter pamelae* strain 7-10-1-b^T^ and *Collinsella aerofaciens* JCM10188^T^. (w = weak, na= no available)

**Figure 4:**
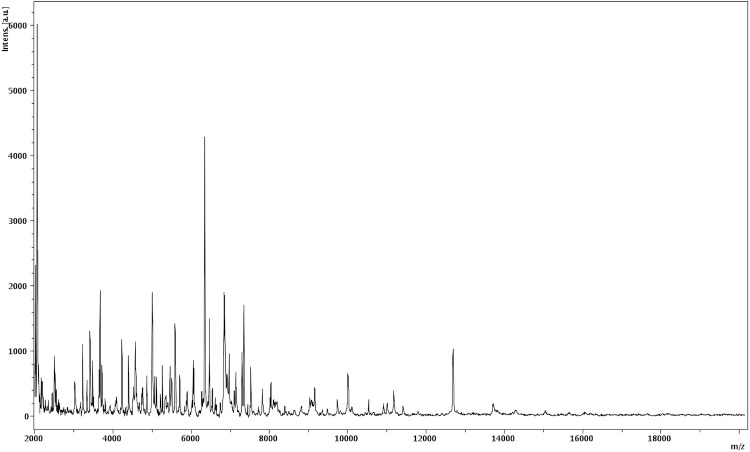
Reference mass spectrum from *S. anaerobia* strain JC110^T^. Spectra from 12 individual colonies were compared and a reference spectrum was generated.

**Figure 5:**
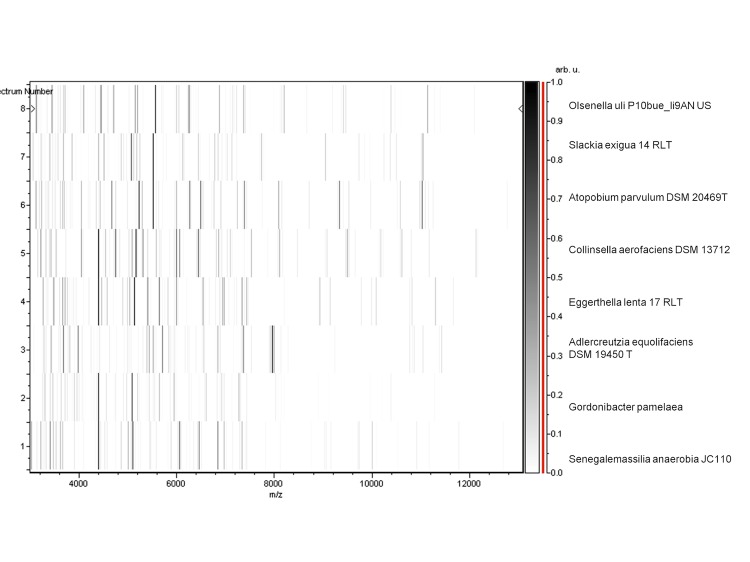
Gel view comparing *Senegalemassilia anaerobia* JC110^T^ spectra with other members into *Coriobacteriaceae* family (*Olsenella uli*, *Slackia exigua, Atopobium parvulum, Collinsella aerofaciens, Eggerthella lenta, Adlercreutzia equolifaciens and Gordonibacter pamelaeae*). The Gel View displays the raw spectra of all loaded spectrum files arranged in a pseudo-gel like look. The x-axis records the m/z value. The left y-axis displays the running spectrum number originating from subsequent spectra loading. The peak intensity is expressed by a Gray scale scheme code. The color bar and the right y-axis indicate the relation between the color a peak is displayed with and the peak intensity in arbitrary units.

Matrix-assisted laser-desorption/ionization time-of-flight (MALDI-TOF) MS protein analysis was carried out as previously described using a Microflex spectrometer (Bruker Daltonics, Germany) [34]. Briefly, a pipette tip was used to pick one isolated bacterial colony from an agar plate, and to spread it as a thin film on an MTP 384 MALDI-TOF target plate (Bruker Daltonics, Leipzig, Germany). Twelve distinct deposits were done for strain JC110^T^ from twelve isolated colonies. Each smear was overlaid with 2 µL of matrix solution (saturated solution of alpha-cyano-4-hydroxycinnamic acid) in 50% acetonitrile, 2.5% tri-fluoracetic-acid, and allowed to dry for five minutes. Measurements were performed with a Microflex spectrometer (Bruker). Spectra were recorded in the positive linear mode for the mass range of 2,000 to 20,000 Da (parameter settings: ion source 1 (IS1), 20 kV; IS2, 18.5 kV; lens, 7 kV). A spectrum was obtained after 675 shots at a variable laser power. The time of acquisition was between 30 seconds and 1 minute per spot. The twelve JC110^T^ spectra were imported into the MALDI BioTyper software (version 2.0, Bruker) and analyzed by standard pattern matching (with default parameter settings) against the main spectra of 3,769 bacteria, which were used as reference data, in the BioTyper database. The method of identification included the m/z from 3,000 to 15,000 Da. For every spectrum, a maximum of 100 peaks taken into account and compared with spectra in the database. A score enabled the identification, of the tested species: a score > 2 with a validly published species enabled the identification at the species level, a score > 1.7 but < 2 enabled the identification at the genus level; and a score < 1.7 did not enable any identification. For strain JC110^T^, no significant score was obtained, suggesting that JC110^T^ was not a member of a known species or genus. We incremented our database with the spectrum from strain JC110^T^ (Figure 4). The gel view allowed us to highlight the spectra differences with other of *Coriobactericeae* family members (Figure 5).

## Genome sequencing information

### Genome project history

The organism was selected for sequencing on the basis of its phylogenetic position and 16S rRNA similarity to other members of the *Coriobacteriaceae*, and is part of a “culturomics” study of the human digestive flora aiming at isolating all bacterial species in human feces. It was the sixth genome of a species within the *Coriobacteriaceae* and the first genome of *Senegalemassilia anaerobia* gen. nov., sp. nov. A summary of the project information is shown in Table 3. The EMBL accession number is CAEM00000000 and consists 8 scaffolds. Table 3 shows the project information and its association with MIGS version 2.0 compliance.

**Table 3:** Project information

**MIGS ID**	**Property**	**Term**
MIGS-31	Finishing quality	High-quality draft
MIGS-28	Libraries used	One paired-end 3 Kb library
MIGS-29	Sequencing platform	454 GS FLX Titanium
MIGS-31.2	Fold coverage	32 ×
MIGS-30	Assembler	Newbler version 2.5.3
MIGS-32	Gene calling method	Prodigal
	EMBL ID	CAEM00000000
	EMBL Date of Release	November 20, 2011
	Project relevance	Study of the human gut microbiome

### Growth conditions and DNA isolation

*Senegalemassilia anaerobia* strain JC110^T^ (= CSUR P147 = DSMZ 25959) was grown on 5% sheep blood-enriched Columbia agar (BioMerieux, Marcy l’Etoile, France) at 37°C in an anaerobic atmosphere. Four petri dishes were spread and resuspended in 3 × 100µl of G2 buffer (EZ1 DNA Tissue kit, Qiagen). A first mechanical lysis was performed using glass powder on a Fastprep-24 device (MP Biomedicals, Ilkirch, France) during 2×20 seconds. DNA was then treated with 2.5 µg/µL lysozyme (30 minutes at 37°C) and extracted through the BioRobot EZ 1 Advanced XL (Qiagen). The DNA was then concentrated and purified on a Qiamp kit (Qiagen). The yield and the concentration was measured by the Quant-it Picogreen kit (Invitrogen) on the Genios_Tecan fluorometer at 25 ng/µl.

### Genome sequencing and assembly

Sequencing was performed using the 3kb paired-end strategy on a Roche 454 Titanium pyrosequencer . This project was loaded twice onto a 1/8 region of a PTP Picotiterplate (Roche, Meylan, France). DNA (5µg) was mechanically fragmented on a Hydroshear device (Digilab, Holliston, MA, USA) with an enrichment size at 3-4 kb. DNA fragmentation was visualized using the Agilent 2100 BioAnalyzer on a DNA labchip 7500 with an optimal size of 3,215 kb. The library was constructed according to the 454 Titanium paired-end protocol. Circularization and nebulization were performed and generated a pattern with an optimum at 363 bp. After PCR amplification through 15 cycles, followed by double size selection, the single stranded paired-end library was quantified using a Quant-it Ribogreen kit (Invitrogen) on the Genios Tecan fluorometer at 152 pg/µL. The library concentration equivalence was calculated to be 7.68E+08 molecules/µL. The library was stored at -20°C until further use.

The library was clonally amplified with 1 cpb in 3 SV-emPCR reactions with the GS Titanium SV emPCR Kit (Lib-L) v2 (Roche). The yield of the emPCR was 12.87%, in the 5 to 20% range from the Roche procedure. Approximately 340,000 beads were loaded onto each of the two 1/8 regions of GS Titanium PicoTiterPlates. Sequencing was performed using the GS Titanium Sequencing Kit XLR70. The runs were performed overnight and then analyzed on the cluster through the gsRunBrowser and Newbler assembler (Roche). A total of 256,934 passed filter wells were obtained and generated 74 Mb of DNA sequence with an average length of 289 bp. The passed filter sequences were assembled using Newbler with 90% identity and 40 bp as overlap. The final assembly yielded 8 scaffolds and 62 large contigs (>1,500 bp).

### Genome annotation

Open Reading Frames (ORFs) were predicted using Prodigal [35] with default parameters. The predicted bacterial protein sequences were searched against the Genbank database and the Clusters of Orthologous Groups (COG) databases using BLASTP. The tRNAScanSE tool [36] was used to find tRNA genes, whereas ribosomal RNAs were found by using RNAmmer [37] and BLASTn against GenBank. ORFans were identified if their BLASTp *E-*value was lower than 1e-03 for alignment length greater than 80 amino acids. If alignment lengths were smaller than 80 amino acids, we used an *E*-value of 1e-05. To estimate the mean level of nucleotide sequence similarity at the genome level between *S*. *anaerobia* and other members of the *Coriobacteriaceae* and among members of this family, we compared genomes two by two and determined the mean percentage of nucleotide sequence identity among orthologous ORFs using BLASTn Orthologous genes were detected using the Proteinortho software [38].

## Genomes properties

**Figure 6:**
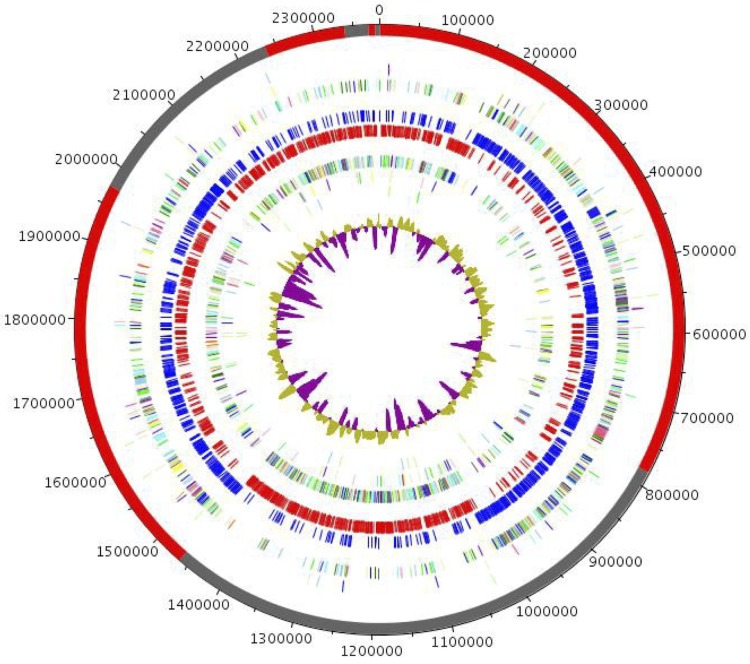
Graphical circular map of the genome from outside to the center: Contigs (red / grey), COG category of genes on the forward strand (three circles), genes on the forward (blue circle) and reverse strands (red circle), COG category on the reverse strand (three circles), GC content.

The genome is 2,383,131 bp long (one chromosome, no plasmid) with a 60.9% G + C content (Table 4). Of the 1,990 predicted genes, 1,932 were protein-coding genes, and 58 were RNAs (1 rRNA operon and 55 tRNA genes). A total of 1,430 genes (68.12%) were assigned a putative function. Fifty-six genes were identified as ORFans (2,90%). The remaining genes were annotated as hypothetical proteins (330 genes = 17.08%). The distribution of genes into COGs functional categories is presented in Table 5 and Figure 6. The properties and the statistics of the genome are summarized in Tables 4 and 5.

**Table 4:** Nucleotide content and gene count levels of the genome

**Attribute**	**Value**	**% of total^a^**
Genome size (bp)	2,383,131	-
DNA coding region (bp)	1,451,434	60.9
DNA G+C content (bp)	2,043,582	85.7
Total genes	1,990	100
RNA genes	58	2.91
Protein-coding genes	1,932	97.09
Genes with function prediction	1,430	74.02
Genes assigned to COGs	1,471	76.13
Genes with peptide signals	205	10.61
Genes with transmembrane helices	463	23.96

The total is based on either the size of the genome in base pairs or the total number of protein coding genes in the annotated genome.

**Table 5:** Number of genes associated with the 25 general COG functional categories

**Code**	**Value**	**%age**	**Description**
J	124	6.42	Translation
A	0	0	RNA processing and modification
K	113	5.84	Transcription
L	97	5.02	Replication, recombination and repair
B	0	0	Chromatin structure and dynamics
D	21	1.09	Cell cycle control, mitosis and meiosis
Y	0	0	Nuclear structure
V	22	1.14	Defense mechanisms
T	74	3.83	Signal transduction mechanisms
M	79	4.09	Cell wall/membrane biogenesis
N	8	0.47	Cell motility
Z	0	0	Cytoskeleton
W	0	0	Extracellular structures
U	34	1.76	Intracellular trafficking and secretion
O	48	2.54	Posttranslational modification, protein turnover, chaperones
C	131	6.78	Energy production and conversion
G	62	3.2	Carbohydrate transport and metabolism
E	192	9.94	Amino acid transport and metabolism
F	53	2.74	Nucleotide transport and metabolism
H	86	4.45	Coenzyme transport and metabolism
I	50	2.59	Lipid transport and metabolism
P	85	4.4	Inorganic ion transport and metabolism
Q	23	1.19	Secondary metabolites biosynthesis, transport and catabolism
R	214	11.08	General function prediction only
S	116	6	Function unknown
-	461	23.85	Not in COGs

## Comparison with *genomes from Coriobacteriaceae*

At present, the complete genomes from *Atopobium parvulum* [39], *Cryptobacterium curtum* [40], *Eggerthella lenta* [41], *Olsenella uli* [42], and *Slackia heliotrinireducens* [43] are available. *S. anaerobia* has a smaller genome than *E. lenta* and *S. heliotrinireducens* (2,384,013 bp *vs* 3, 632,260 bp and 3,165,038 bp, respectively) but larger than *A. parvulum*, *C. curtum*, and *O. uli* (2,384,013 bp *vs* 1,543,805 bp, 1,617,804 bp, and 2,051,896 bp, respectively). It has a greater number of genes than *A. parvulum*, *C. curtum* and *O. uli* (1,900 *vs* 1,419, 1,425 and 1,850 genes, respectively) but fewer than *E. lenta* and *S. helionitrireducens* (3,181 and 2,858 genes, respectively), and has a higher G+C content than *A. parvulum, C. curtum* and *S. heliotrinireducens* (60.9% *vs* 45.69% and 50.91%, 60.21%, respectively) but smaller than *E. lenta* and *O. uli* (64.2% and 64.7%, respectively). Table 6 summarizes the numbers of orthologous genes and the average percentage of nucleotide sequence identity between the different genomes studied. The average nucleotide identity ranged from 47.74 to 71.10% within the *Coriobacteriaceae* family, and from 47.74% to 67.05% between *S. anaerobia* and other species.

**Table 6:** Number of orthologous genes (upper right) and average nucleotide identity levels (lower left) between pairs of genomes determined using the Proteinortho software [38].

	*S. anaerobia*	*S. heliotrinireducens*	*O. uli*	*E. lenta*	*C. curtum*	*A. parvulum*
*Senegalemassilia anaerobia*	-	962	471	1,059	877	625
*Slackia heliotrinireducens*	66.94	-	715	1,019	832	646
*Olsenella* *uli*	67.05	67.49	-	736	611	694
*Eggerthella lenta*	47.74	71.10	68.69	-	908	670
*Cryptobacterium curtum*	64.78	65.88	63.12	66.74	-	606
*Atopobium parvulum*	62.76	62.51	65.77	61.52	63.87	-

## Conclusion

On the basis of phenotypic (Table 2), phylogenetic and genomic analyses (Table 6), we formally propose the creation of *Senegalemassilia anaerobia gen. nov.,* sp. nov. that contains strain JC110^T^. This bacterium has been found in Senegal.

### Description of *Senegalemassilia gen. nov.*

*Senegalemassilia* (se.ne.ga.le.ma.si’li.a N.L. fem. N. Senegalemassilia, combination of Senegal, where the stool was collected and massilia, the latin name of Marseille, where strain JC110^T^ was cultivated.)

Gram-positive coccobacilli. Strictly anaerobic. Mesophilic. Motile. Absent catalase, oxydase and indole productions. Positive for arginine dihydrolase, nitrate reduction and alkanine phosphatase. Habitat: human digestive tract. Type species: *Senegalemassilia anaerobia*.

### Description of *Senegalemassilia anaerobia sp. nov.*

*Senegalemassilia anaerobia* (an.a.e.ro’bi.a. N. L. F. adj. Gr. pref. *an* not; Gr. N. *aer* air; Gr.n.*bios* life; N.L. adj. *anaerobia* anaerobe, that can live in the absence of oxygen; referring to the respiratory metabolism of organism). It has been isolated from the feces of an asymptomatic Senegalese patient.

Gram-positive coccobacilli, 0.7 µm in diameter and 1.56µm in length. Strictly anaerobic. Mesophilic. Motile and non-sporulating. Colonies are transparent and smooth with 0.5 mm in diameter on blood-enriched Columbia agar. Catalase oxydase and indole negative. Arginine dihydrolase, nitrate reduction,alkanine phosphatase, acid phosphatase and Naphtlol-AS-BI-phosphohydrolase positive. Asaccharolytic. Cells are susceptible to amoxicillin, imipenem, metronidazole and gentamicin but resistant to trimethoprim/sulfamethoxazole. The 16S rRNA and genome sequences are deposited in Genbank and EMBL under accession numbers JF824809 and CAEM00000000, respectively. The G+C content of the genome is 60.9%. Habitat: human digestive tract. The type strain JC110^T^ (= CSUR P147 = DSMZ 25959) was isolated from the fecal flora of a healthy patient in Senegal.
